# Honoring the voices of bereaved caregivers: a Metasummary of qualitative research

**DOI:** 10.1186/s12904-017-0231-y

**Published:** 2017-09-06

**Authors:** Lorraine Holtslander, Sharon Baxter, Kelly Mills, Sarah Bocking, Tina Dadgostari, Wendy Duggleby, Vicky Duncan, Peter Hudson, Agatha Ogunkorode, Shelley Peacock

**Affiliations:** 1 College of Nursing, University of Saskatchewan, Rm 4216, E-Wing Health Sciences, 104 Clinic Place, Saskatoon, SK S7N 2Z2 Canada; 2 University of the Witwatersrand, Johannesburg, South Africa; 3 Executive Director of the Canadian Hospice Palliative Care Association, Ottawa, ON Canada; 4 RA; College of Education, University of Saskatchewan, Saskatoon, SK Canada; 5 RA, College of Nursing, University of Saskatchewan, Saskatoon, SK Canada; 6 RA, University of Saskatchewan, Saskatoon, SK Canada; 7 Faculty of Nursing, University of Alberta, Edmonton, AB Canada; 8 University of Saskatchewan, Saskatoon, SK Canada; 9 Palliative Care c/o St. Vincent’s Hospital and Collaborative Centre of the University of Melbourne, Melbourne, Australia; 10 Palliative Care, Queen’s University, Northern Ireland, UK; 11 College of Nursing, University of Saskatchewan, Saskatoon, SK Canada; 12 College of Nursing, University of Saskatchewan, Saskatoon, SK Canada

**Keywords:** Bereavement, Family caregiver, Palliative care, Qualitative, Metasummary, Support

## Abstract

**Background:**

Family caregiving in the context of advanced disease in particular, can be physically and emotionally taxing. Caregivers can subsequently face bereavement exhausted with few supports, limited resources and a significant proportion will develop negative psychological and social outcomes. Although some research has attended to the bereavement experiences of family caregivers who had cared for a person requiring palliative care, a comprehensive qualitative understanding of the impact of caregiving on bereavement has not been articulated. The purpose of this study was to conduct a qualitative metasummary to explore the experiences of bereaved family caregivers of people who received palliative care services, regardless of their underlying disease.

**Methods:**

Sandelowski and Barroso’s qualitative metasummary method was utilized: 1287 articles were identified through extensive database searches (i.e. – MEDLINE, PsychINFO, and CINAHL) and reviewed to determine if they fit the criteria. Those included in the review were assessed for study quality. Findings from each study were then thematically coded and a frequency of themes was calculated.

**Results:**

The sample consisted of 47 qualitative studies. A total of 15 themes emerged. In descending order of frequency, the 15 themes were: the individual emotions of serenity, sadness, guilt, uncertainty, trauma, escape, and anger; post-loss experiences that helped the caregiver in bereavement; post-loss experiences that hindered; practical life changes; caregiver role identity; pre-loss experiences that helped; pre-loss experiences that hindered; caregiver context; and a need for different kinds of supports. Three key findings emerged from the themes: (1) many different aspects of the caregiving experience impact the bereavement experience, (2) every bereavement experience is unique, and (3) a variety of supports must be developed and made available to caregivers to meet these unique needs.

**Conclusions:**

Based on the metasummary findings, changes are needed in practice and policy to ensure the health and well-being of the family caregiver is maintained by offering support both during caregiving and bereavement.

## Background

While acknowledging that most of the world has inadequate palliative care services [1] and that considerable research has explored family caregivers’ experiences while receiving palliative care, there appears to be few investigations that follow the experience of family caregivers of palliative care patients into bereavement. Most studies focus on caregivers’ experiences of active caring work [2], and few explore how these individuals’ bereavement trajectories might differ from the general population. Since caregivers often provide care to persons who are terminally ill, they are frequently in contact with professional palliative care services during active caregiving. Although such services are mandated to provide psychosocial support to family caregivers that extends into bereavement, by both national [3] and international [4] palliative care guidelines, research suggests that in reality, aid is rarely made available in a systematic and evidence based way to family caregivers after the patient dies [5]. Palliative care services face a number of obstacles in providing adequate bereavement care, including immense paperwork in providing follow-up and a lack of funding [6]. Researchers have suggested that bereavement support has been deficient and remains the least well-developed aspect of hospice and specialist palliative care services [7].

In the pre-death period, caregivers of palliative care patients, by virtue of their role, are situated in close proximity to the care recipients’ dying process and this caring work often takes a mental, emotional, and physical toll on the caregiver [2]. Accordingly, it seems that caregivers face unique challenges and outcomes in bereavement, and, without adequate supports, are a particularly vulnerable group [8]. Caregivers’ experiences in the pre-death period have been shown to influence their grief reactions in different ways. Indeed, quantitative researchers have correlated factors like the patient’s symptom severity [9], the use of aggressive end-of-life treatments like resuscitation [10], the patient’s place of death and the type and intensity of emotions caregivers experience post-loss [11] with poorer bereavement outcomes.

Although research suggests that most caregivers experience a decline in depressive symptoms over the course of bereavement [12], other work demonstrates that many factors can predispose caregivers to complicated or prolonged grief [13]. In one study, complicated grief presented in nearly one third of caregivers even after more than a year post-bereavement [14]. Caregivers who experience persistent difficulties with grief, as a result of their role, therefore present a significant public health issue. Given the gaps in service-delivery and research related to bereaved caregivers, it is vital that the experiences of this population be investigated more thoroughly. By exploring their experiences of losing the person they had cared for, caregivers’ bereavement needs may be given a voice, and ultimately more effective, evidence-based approaches to bereavement care may be developed.

The purpose of this study was to explore the experiences of bereaved family caregivers who had received palliative care services, by completing a metasummary of the qualitative research in this area in order to develop a full explanation of their experiences of bereavement after caregiving.

## Methods

Given the wide range of qualitative research focused specifically on bereaved family caregivers who interacted with the palliative care system, metasummary methods were chosen to answer the research question. Sandelowski and Barroso [15] developed the method known as metasummary to equip researchers with an effective way to integrate and summarize qualitative research findings. Metasummary is a particularly useful tool for bringing together and interpreting qualitative findings with varying levels of researcher interpretation, including topical or thematic surveys [16]. Although it is a tool for working with qualitative research, metasummary is primarily a quantitatively oriented approach, which aims to discern the frequency with which different findings have been reported across an aggregation of studies on a common topic. Sandelowski and Barroso [15] argue that these numbers have no meaning on their own, but provide a way to assess the relative frequency of a finding across the reports included in the metasummary.

### Literature search

Qualitative research on the bereavement experiences of family caregivers was searched for in the following databases: MEDLINE (from inception to July 15, 2014), Embase (from inception until July 17, 2014), and PsycINFO (from inception until July 17, 2014). The first three databases were searched via the OVID interface. Additional databases searched were CINAHL (from inception until June 11, 2014, Ebsco interface), Scopus (from inception until September 17, 2014), Web of Science (from inception until September 17, 2014), Academic Search Premier (from inception until September 17, 2014), the Cochrane Central Register of Controlled Trials (CENTRAL) in the Cochrane Library (September 17, 2014), the Joanna Briggs Institute EBP Database (September 17, 2014), and Ageline (from inception to September 17, 2014). The Proquest Dissertations and Theses database was searched for dissertations (from inception to September 17, 2014).

The MEDLINE database search strategy was developed by a librarian experienced in systematic review searching. Research team members provided feedback for the initial search strategy, and amendments were made to optimize the search results. The MEDLINE search was adapted for the other databases. The MEDLINE search strategy is available in Appendix 1. Because the focus of the research project was to explore the experiences of bereaved family caregivers of persons with terminal illness who received palliative care, only qualitative and mixed methods studies were included. The librarian used the University of Texas School of Public Health filter for retrieving qualitative studies.

The research team agreed to the following inclusion criteria for the metasummary: (1) qualitative or mixed methods research studies dated between 1990 and 2014, (2) of the experiences of bereaved family caregivers for a person who was 18 years of age and older and (3) were receiving palliative care services. Additional criteria included English language studies from any country and both published and unpublished studies, such as theses. Search strategies were saved, and set up as “alerts” which notified the team when new articles were published. To ensure identification of all relevant studies, the reference lists of included studies or relevant reviews were scanned. Authors responsible for publications meeting the inclusion criteria were contacted and asked if they had additional articles accepted for publication.

### Literature appraisal

Studies that met the inclusion criteria were appraised for overall research quality using the Critical Appraisal Skills Programme (CASP) tool [17]. The CASP consists of a series of questions researchers may use to systematically assess different dimensions of a study’s methodological quality, including reliability, degree of bias, and significance [17]. In the series of questions developed for assessing qualitative research specifically, two binary choice (yes/no) questions are followed by eight ordinal questions that ask the rater to award the article 1 to 3 points. Values for the latter questions are summed (maximum total for each article is 24 points) and the resulting total serves as a measure of the article’s overall quality [18]. Each study included in the final sample was appraised with the CASP by a minimum of two independent raters. Raters’ values were then compared, discrepancies were identified, and reasons for discrepancies were discussed by the two raters and the principal investigator. Conclusive values were negotiated and assigned during these discussions.

### Literature classification

The methods section of each article was reviewed. Based on the investigators’ reported methods for data collection and analysis, each study was categorized as employing one of six qualitative research methods: content analysis, grounded theory, phenomenology, interpretive description, mixed methods or ethnography. Sandelowski and Barroso’s [19] typology of qualitative research findings was used to categorize the studies further. Sandelowski and Barroso devised four major categories of findings, situated on a continuum from least to most interpretive: topical survey, thematic survey, conceptual thematic description, and interpretive explanation. Each study was evaluated and coded using this framework.

### Data analysis

The findings of all included research articles were analyzed by adapting the steps of metasummary data analysis as described by Sandelowski, Barrosso, and Voils [16]: (a) extraction, (b) grouping, (c) abstraction, (d) formatting, and (e) a calculation of the frequency of each finding. The following will be a discussion of how each of these steps were carried out.

#### Extraction and grouping

All research articles were imported into the coding program NVIVO Version 11 (QSR International, 2015) for analysis. Only the findings of each research report were subjected to analysis, as the aim of the metasummary was to integrate and summarize original results generated by each report (rather than background or methodological information). However, the findings of a study are not always compartmentalized in a distinct section of a research report. As such, text that qualifies as findings (based on chosen criteria) must be located and extracted from each report [16].

In the present study, findings were defined as any segment of text that was (1) descriptive of the experiences of family caregivers and (2) part of or based on the study’s original data. Findings did not include references to other studies (e.g. literature reviews), descriptions of a study’s methods, or discussions of a study’s significance. The criteria used for data extraction in the present study diverged from Sandelowski, Barroso, and Voils [16] in that “raw” data - participant experiences expressed in their own words - were considered findings in themselves and were also extracted. This decision was made with the intent to reach a comprehensive understanding of bereaved family caregivers’ experiences, as described not only by researchers but also by family caregivers themselves.

Three independent researchers located and extracted the findings from the research reports. At the time of extraction, each finding was also grouped or coded according to emerging similarities in content. The coding process was highly iterative and involved many discussions between coders throughout data analysis. Midway through the coding process, the research team also invited community members – including bereaved caregivers from a local community agency and helping professionals such as palliative care doctors and social workers – to a public research forum. The emerging themes (and sampled quotes from articles included in the metasummary) were shared in this public research forum in a roundtable format, and feedback was elicited from all guests. These discussions were used to further refine the codes. The final codes divided findings into themes and subthemes, ensuring that the data would be manageable while retaining the complexity of the findings.

#### Abstraction and formatting

After the final groupings of themes and subthemes were devised, each theme was titled with a phrase that captured its general content and a description of each theme was composed. As in the grouping stage of data analysis, abstraction was a highly collaborative process. Three researchers and the principal investigator regularly met to evaluate and reflect upon the findings, and all contributed to the final titles and descriptions. Descriptions aimed to succinctly summarize the most prominent findings of each theme, as well as the relationships between themes. Following the steps of data abstraction and formatting, three overarching findings or meta-themes about caregiver experiences, relevant to future research and policy, were identified.

#### Frequency of themes calculation

In order to ascertain the themes and subthemes that were most prevalent in the literature sample, the frequency was calculated for each theme and subtheme. The total number of studies containing a certain finding was divided by the total number of reports in the metasummary sample. Frequency of each subtheme was also calculated, but for the sake of conciseness, within each theme only the three subthemes with the highest frequency is included in the main report. A complete list of themes and subthemes is available on request.

## Results

**Fig. 1:**
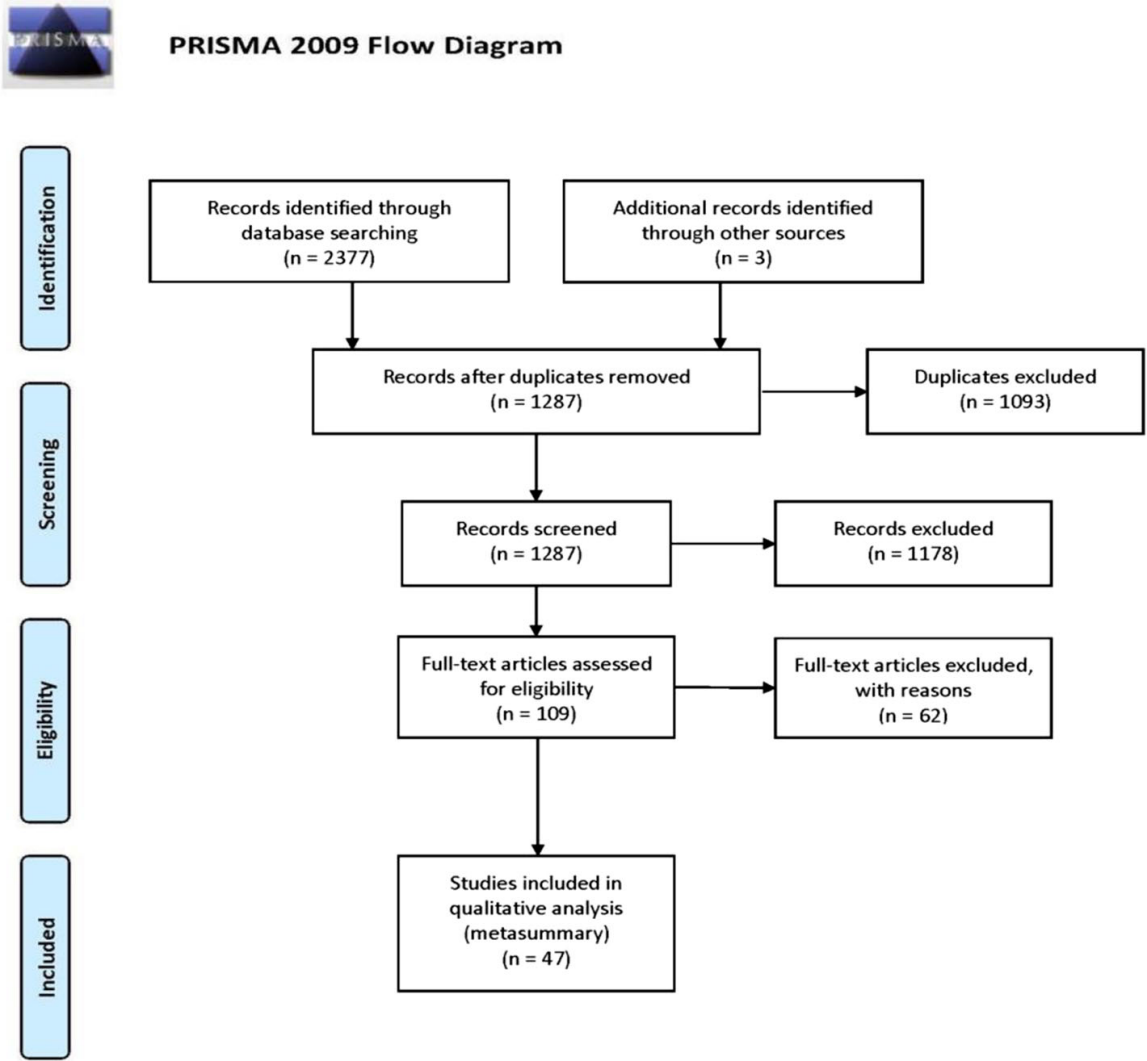
PRISMA flow chart search results. PRISMA: Preferred Reporting Items for Systematic Reviews and Meta-Analyses

Figure 1 visualizes the flow of articles included and excluded at each stage of the metasummary process. In total, 2377 records were identified through the database search, and entered in EndNote 7.0 software (Thomson Reuters, Philadelphia, USA), a software-based reference management system, resulting in 1284 after duplicate references were removed. The team evaluated the studies for inclusion using DistillerSR software (Evidence Partners, Ottawa, Canada), ensuring two members of the research team independently screened each title, abstract, and full-text article. Both reviewers were required to agree and all conflicts were flagged for a third reviewer. The final sample was discussed with the research team to ensure they met the inclusion criteria. A total of 47 studies were included for analysis.

Study characteristics including CASP score, methodology, country, patient disease, and caregiver qualities (age, gender, and relationship to patient) were extracted from each article and are presented in Table 1. The CASP scores of the sampled articles ranged from 11 to 22 with a mean score of 18.6. Sampled studies used a number of methodologies: grounded theory (13), content analysis (11), mixed methods (9), phenomenology (9), interpretive description (3), and ethnography (2). Sampled studies used a number of analysis methods: conceptual thematic description (20), interpretation (17), topical survey (3), thematic survey (7), and topical survey (3). The studies were conducted in various countries including: the USA (14), Canada (11), Australia (7), the United Kingdom (4), Sweden (4), Japan (2), Hong Kong (2), New Zealand (1), Republic of Korea (1), and Denmark (1).

**Table 1:** Characteristics of the Studies included in the Metasummary

Source	Country	Method- ology	Data Collection Method	CASP Score	Finding Classification	Gender of Participants (N=)	Relationship to the care receiver	Caregiver age range (years); Caregiver mean age	Time since bereavement; Average bereavement period
Agnew et al. (2008)	Northern Ireland	Content Analysis	Semi-structured interviews	21	Conceptual / Thematic Description	Male = 5; Female = 5	Spouses = 10; Parents = 0 Children = 0; Sibling = 0; Other = 0	39 years - 64 years; 52 years	13 months - 23 months; not reported
Almberg et al. (2000)	Sweden	Field Research	Interviews consisted of broad open-ended questions, follow-up questions, and field notes	20	Conceptual/ Thematic Description	Male = 9; Female = 21	Spouses = 7; Parents = 0 Children = 18; Sibling = 3; Other = 2	49 years - 88 years; 71 years	3 months - 5 months; 3.33 months
Aoun et al. (2012)	Australia	Mixed methods Other: Thematic Analysis	Demographic questionnaire, semi-structured interview, and a measure of prolonged grief.	18	Thematic survey	Male = 3; Female = 13	Spouses = 16; Parents = 0 Children = 0; Sibling = 0; Other = 0	50 years - 82 years; 65.19 years	1 year - 4 years; 27.5 months
Asai et al. (2010)	Japan	Content Analysis	Semi-structured interviews	16	Thematic Survey	Male = 7; Female = 17	Spouses = 24; Parents = 0 Children = 0; Sibling = 0; Other = 0	36 years −71 years; 59 years	2 months - 9 years; 4 years
Bellamy (2014)	New Zealand	Grounded Theory	Semi-structured telephone interview and short background questionnaire	21	Conceptual/ Thematic Description	Male = 9; Female = 19	Spouses = 17; Parents = 4 Children = 4; Sibling = 0; Other = 3	71 years - 90 years; not reported	within 1 year; not reported
Benkel et al. (2009)	Sweden	Mixed Methods	Semi-structured questionnaire with the possibility of giving comments, in-depth interview, and the Sense of Coherence Scale	16	Topical survey	Male = 1; Female = 6	not reported	not reported; not reported	6 weeks - 8 weeks and at 1 year; not reported
Bennett (2009)	England	Grounded Theory	Semi-structured interview	18	Conceptual/ Thematic Description	Male = 46; Female = 45	Spouses = 91; Parents = 0 Children = 0; Sibling = 0; Other = 0	55 years - 95 years; 74 years	3 months - 32 years; 8.68 years
Bent & Magilvy (2006)	USA	Phenomenology	In-depth open ended questions in interview and field notes	18	Conceptual / Thematic Description	Men = 0; Women = 6	Spouses = 6; Parents = 0 Children = 0; Sibling = 0; Other = 0	50 years - late 70s; not reported	1 year - 36 years; not reported
Cadell (2007)	Canada	Grounded Theory	Semi-structured interview	18	Interpretive	Male = 8; Female = 4; Transgender = 3	not reported	not reported; not reported	not reported; not reported
Chan et al. (2011)	Hong Kong	Grounded Theory	Interviews	20	Interpretive	Male = 5; Female = 10	Spouses = 15; Parents = 0 Children = 0; Sibling = 0; Other = 0	66 years - 86 years; 74.4 years	6 weeks; 6 weeks
Chan et al. (2013)	Hong Kong	Other: Qualitative Content Analysis	Semi-structured interview and field notes	19	Thematic Survey	Male = 5; Female = 5	Spouses = 0; Parents = 0 Children = 10; Sibling = 0; Other = 0	22 years - 46 years; not reported	not reported; not reported
Dean et al. (2005)	Canada	Content Analysis	Semi-structured interview and field notes	17	Interpretive	Male = 4; Female = 9	Spouses = 0; Parents = 0 Children = 13; Sibling = 0; Other = 0	40 years - 77 years; 62.1 years	6 months - 6 years; 28.6 months
Denham (1999)	USA	Ethnography	Semi-structured interviews, observation, patient record review and field notes	21	Interpretive	not reported (“8 families”)	Spouses = 0; Parents = 0 Children = 0; Sibling = 0; Other = 29	54 years - 71 years; not reported	within 6 months; not reported
DiGiacomo et al. (2013)	Australia	Interpretative Phenomenological Analysis (IPA)	Three in-depth semi-structured interviews	21	Interpretive	Male = 21; Female = 0	Spouses = 21; Parents = 0 Children = 0; Sibling = 0; Other = 0	63 years - 82 years; 71.4 years	within 2 years; not reported
Duke (1998)	England	Phenomenology	Unstructured interviews	21	Interpretive	not reported	Spouses = 4; Parents = 0 Children = 0; Sibling = 0; Other = 0	not reported; not reported	2 years; 2 years
Dumont et al. (2008)	Canada	Content Analysis	Semi-structured interviews	18	Conceptual / Thematic Description	Male = 7; Female = 11	Spouses = 12; Parents = 0 Children = 0; Sibling = 0; Other = 6	33 years - 75 years; not reported	3 months −4 months; not reported
Ferrell & Boyle (1992)	USA	Field Research	Interview with focused questions to obtain demographic information and open-ended techniques to gather data	16	Conceptual/ Thematic description	Male = 5; Female = 0	Spouses = 5; Parents = 0 Children = 0; Sibling = 0; Other = 0	25 years - 45 years; not reported	not reported; not reported
Fisker & Strandmark (2007)	Denmark	Descriptive Phenomenology	In-depth interview with interview guide containing open themes	22	Interpretive	Male = 2; Female = 6	Spouses = 8; Parents = 0 Children = 0; Sibling = 0; Other = 0	52 years - 69 years; not reported	6 months - 18 months; not reported
Grbich et al. (2001)	Australia	Interpretive	Open-ended questions in interviews	18	Conceptual / Thematic Description	Male = 9; Female = 11	Spouses = 15; Parents = 0 Children = 5; Sibling = 0; Other = 0	49 years - 83 years; not reported	not reported; not reported
Hegge (1991)	USA	Content Analysis	Open-ended questions in interviews	11	Thematic Survey	Male = 5; Female = 21	Spouses = 26; Parents = 0 Children = 0; Sibling = 0; Other = 0	over 60 years; not reported	0 years −3 years; not reported
Holtslander & Duggleby (2009)	Canada	Grounded Theory	Open-ended questions in interview, diaries, field notes and memos	21	Interpretive	Male = 0; Female = 13	Spouses = 13; Parents = 0 Children = 0; Sibling = 0; Other = 0	60 years - 79 years; not reported	3 months - 1 year; not reported
Holtslander & Duggleby (2010)	Canada	Grounded Theory	Open-ended questions in interview and diaries	21	Interpretive	Male = 0; Female = 13	Spouses = 13; Parents = 0 Children = 0; Sibling = 0; Other = 0	60 years - 79 years; 72.6 years	0–1 year; not reported
Holtslander et al. (2011)	Canada	Grounded Theory	In-depth interviews, journal entries, field notes, memos	18	Interpretive	Male = 3; Female = 7	Spouses = 10; Parents = 0 Children = 0; Sibling = 0; Other = 0	66 years - 83 years; not reported	2 months - 11 months; 6.1 months
Hornjatkevyc & Alderson (2011)	Canada	Phenomenology	Semi-structured interviews	20	Conceptual / Thematic Description	Male = 8; Female = 0	Spouses = 8; Parents = 0 Children = 0; Sibling = 0; Other = 0	44 years - 53 years; not reported	18 months - 9 years; not reported
Hudson (2006)	Australia	Mixed Methods	Questionnaires and structured interviews	14	Conceptual / Thematic Description	Male = 14; Female = 31	Spouses = 28; Parents = 0 Children = 0; Sibling = 0; Other = 17	not reported; 58 years	6 weeks; 6 weeks
Jacob (1996)	USA	Grounded Theory	Semi-structured interview	19	Interpretive	Male = 0; Female = 6	Spouses = 6; Parents = 0 Children = 0; Sibling = 0; Other = 0	66 years - 78 years; 74.3 years	6 weeks - 16 months; not reported
Jones & Martinson (1992)	USA	Grounded Theory	Open-ended interview and semi-structured interview	12	Conceptual/Thematic Description	Male = 4; Female = 9	Spouses = 7; Parents = 0 Children = 6; Sibling = 0; Other = 0	38 years - 78 years; 58.8 years	1 month - 20 months; 8.15 months
Kerr (1991)	USA	Mixed Methods	Structured interview with some open-ended questions, two scales	21	Thematic survey	Male = 0; Female = 67	Spouses = 0; Parents = 0 Children = 67; Sibling = 0; Other = 0	35 years - 69 years; 48.7 years	not reported; not reported
Koop & Strang (2003)	Canada	Content Analysis	Interviews, field notes and observation	19	Conceptual / Thematic Description	Male = 4; Female = 11	Spouses = 9; Parents = 0 Children = 5; Sibling = 1; Other = 0	37 years - 81 years; 58.5 years	1 month - 12 months; 5.3 months
Lee et al. (2005)	Republic of Korea	Mixed Methods	In-depth semi-structured interviews, observation, and two instruments on grief	15	Thematic Survey	Male = 5; Female = 5	Spouses = 10; Parents = 0 Children = 0; Sibling = 0; Other = 0	36 years −57 years; 49.1 years	10 months - 28 months; 16.2 months
Longman (1995)	USA	Grounded Theory	Interviews; questions moved from general to specific. Memos and descriptive field notes.	18	Interpretive	Male = 0; Female = 6	Spouses = 0; Parents = 6 Children = 0; Sibling = 0; Other = 0	45 years - 69 years; 56.5 years	10 months - 4 years; not reported
McGaffic & Longman (1993)	USA	Grounded Theory	Early interviews began with one question; later interviews contained more focused questions	17	Interpretive	Male = 6; Female = 0	Spouses = 6; Parents = 0 Children = 0; Sibling = 0; Other = 0	24 years - 48 years; 39 years	3 weeks - 18 months; 6.1 months
Meares (1995)	USA	Descriptive Phenomenology	Semi-structured interview, interview observations, field notes, and personal researcher log	24	Interpretive	Male = 0; Female = 12	Spouses = 3; Parents = 4 Children = 0; Sibling = 3; Other = 2	40 years - 75 years; 61.1 years	2 months - 14 months; 5.83 months
Milberg et al. (2008)	Sweden	Mixed Methods	Postal questionnaire with Likert-type and open-ended questions	18	Topical Survey	Male = 99; Female = 147; unknown = 2	Spouses = 132; Parents = 54; Children = 37; Sibling = 0; Other = 21	27 years - 93 years; 62 years	0 months - more than 6 months; not reported
Muta et al. (2014)	Japan	Other: Content Analysis	Semi-structured interview	20	Topical survey	Male = 13; Female = 31	Spouses = 20; Parents = 0; Children = 18; Sibling = 3; Other = 3	21 years - 79 years; 58 years	18 months - 26 months; 22 months
O’Callaghan et al. (2013)	Australia	Grounded Theory	Questionnaire and semi-structured interviews	16	Conceptual / Thematic Description	Men = 3; Women = 5	Spouses = 2; Parents = 2 Children = 2; Sibling = 1; Other = 2	21–70 years; not reported	18 days - 3 years; 1 year
Pusa et al. (2012)	Sweden	Phenomenology	Narrative interviews	20	Interpretive	Men = 2; Women = 9	Spouses = 7; Parents = 0 Children = 3; Sibling = 0; Other = 1	35–79 years; 57.9 years	3 months −11 months; 8 months
Rafieei (2013)	USA	Comparative Analysis	Semi-structured interview	22	Conceptual/ Thematic Description	Male = 24; Female = 24	Spouses =48; Parents = 0 Children = 0; Sibling = 0; Other = 0	40 years and older; not reported	0 years - more than 3 years; not reported
Sanderson et al. (2013)	Australia	Mixed method	Semi-structured phone interview	19	Thematic Survey	Men = 20; Women = 12	Spouses = 20; Parents = 0 Children = 8; Sibling = 4; Other = 0	31 years - 81 years; 58 years	6 months; 6 months
Shuter et al. (2014)	Australia	Other: Problem-Driven Content Analysis	Semi-structured interview	21	Conceptual/ Thematic Description	Male = 3; Female = 4; unknown = 6	Spouses = 7; Parents = 0 Children = 6; Sibling = 0; Other = 0	not reported; 67.1 years	pre-death - 12 months; not reported
Small et al. (2009)	United Kingdom	Thematic Analysis	Semi-structured interview (face to face or telephone)	19	Conceptual/ Thematic Description	Male = 3; Female = 17	Spouses = 13; Parents = 0 Children = 7; Sibling = 0; Other = 0	not reported; not reported	0 months - 18 months; not reported
Sowell et al. (1991)	USA	Descriptive Phenomenology	Open-ended interview	21	Conceptual/ Thematic description	Male = 8; Female = 0	Spouses = 8; Parents = 0 Children = 0; Sibling = 0; Other = 0	26 years - 53 years; not reported	0 months - 18 months; not reported
Stajduhar (1997)	Canada	Grounded Theory	Open-ended interview, observational field notes, theoretical memos and diagrams, and personal researcher journal	19	Interpretive	Male = 4; Female = 3	Spouses = 4; Parents = 2 Children = 0; Sibling = 1; Other = 0	31 years - 65 years; not reported	within 1 year (except 1); not reported
Stajduhar et al. (2010)	Canada	Interpretive thematic	Focus groups	14	Interpretive	Males = 5; Females = 14	Spouses = 13; Parents = 0 Children = 4; Sibling = 1; Other = 1	42 years - 85 years; 63 years	2 years −11 years; not reported
Steeves (2002)	USA	Ethnography	Serial interviews and participant observation and field notes	20	Conceptual / Thematic Description	Male = 5; Female = 10	Spouses = 15; Parents = 0 Children = 0; Sibling = 0; Other = 0	not reported; 70.3 years	pre-death - 29 months; not reported
Topf et al. (2013)	Canada	Interpretive Descriptive	Semi-structured interviews, field notes, journals	17	Conceptual/ Thematic Description	Males = 5; Females = 13	Spouses = 8; Parents = 1 Children = 4; Sibling = 3; Other = 2	23–91 years old; not reported	2 months - 17 years; 3.96 years
Waldrop (2007)	USA	Exploratory Descriptive Phenomenological	Two open-ended interviews, Brief Symptom Inventory, and Texas Revised Instrument on Grief	19	Thematic survey	Male = 7; Female = 23	not reported	61 years - 85 years; 77 years	1 year; 1 year

In total, 1132 participants who were bereaved after caregiving were included in the metasummary. Caregiver age ranged from 21 to 95 with a mean of 62.68 years although not all studies reported the caregiver age, gender, relationship to the patient, or illness causing bereavement Most caregivers were reported as female (*n* = 701 or 62%), 396 were reported as male (35%) and 3 as transgender (0.2%). Sampled caregivers had various relationships to the deceased: 687 (61%) were spouses or partners, 344 (30%) were relatives (parent, parent-in-law, child, sibling, aunt, grand-daughter, daughter-in-law, “other family” or “family”), 20 (2%) were friends, and 24 (2%) were ‘other’ or not clearly specified. Of the 344 relatives, 204 were children or children-in-law (18%), 86 (8%) were parents or parents-in-law and 20 were siblings (2%).

In the sample, 651 participants were bereaved by cancer (58%), 59 were bereaved by dementia/Parkinson disease (5%), 32 were bereaved by cardiovascular or respiratory disease (3%), 3 were bereaved by stroke (0.3%), and 5 (0.4%) were bereaved by other causes (“other”, accident, infection). The health condition leading to bereavement for 270 (24%) participants was not clearly defined. The duration of caregiving was reported by 12 of the 47 studies and ranged from less than 1 month to 13 years. When reported, at the time of data collection, the period of caregiver bereavement had ranged from less than a year to 9 years.

### Themes

The aggregated findings from the data sample were combined into broader themes and subthemes. A total of 15 themes emerged from the analysis to represent all of the data, with seven of those themes grouped under the meta-theme of emotional journeys and eight themes standing alone. Themes are ordered according to frequency. Frequency calculations for each theme are presented in Table 2.

**Table 2:** Frequency of Themes

Themes and subthemes	Frequency (%) and(n)	Citation of articles including this finding
**Emotions**	**89.4 (42)**	[20–29, 31–41, 43–51, 53, 55–57, 65–72]
**Serenity**	**74.5 (35)**	[20–29, 31–36, 38, 40, 41, 44–49, 53, 55–57, 65–67, 69, 71, 72]
**Sadness**	**72.3 (34)**	[20–27, 31–33, 35–41, 43–49, 51, 53, 55, 57, 65–67, 70–72]
**Guilt**	**53.2 (25)**	[20, 22–27, 32, 34, 37, 41, 43–47, 49, 50, 55–57, 68–71]
**Uncertainty**	**53.2 (25)**	[20, 22–26, 31–33, 35, 36, 39–41, 43–45, 48, 49, 53, 56, 67, 69, 71, 72]
**Trauma**	**40.4 (19)**	[20, 21, 23, 32, 37, 39, 41, 44–46, 48, 49, 53, 56, 57, 65, 67, 68, 71]
**Escape**	**36.2 (17)**	[22, 23, 25, 31, 32, 34, 36, 39, 41, 45, 46, 49, 53, 57, 66, 71, 72]
**Anger**	**31.9 (15)**	[23, 25, 31–33, 37, 40, 41, 44, 47, 49, 51, 57, 66, 67]
**Post-loss help**	**87.2 (41)**	[20, 22–51, 53, 55, 57, 65–67, 69, 70, 72, 73]
Family and friends	61.7 (29)	[20, 22, 23, 25–41, 43, 44, 47, 49, 53, 65, 67, 72, 73]
Formal support	42.6 (20)	[23, 26, 30–34, 37, 38, 40–44, 46, 47, 50, 65, 70, 73]
Coping strategies	38.3 (18)	[23, 24, 31–37, 40, 41, 44, 47, 49, 51, 55, 65, 66]
**Post-loss hinder**	**74.5 (35)**	[20–23, 25–30, 32–34, 36–49, 51–53, 65, 67, 70–72]
Family and friends - negative	46.8 (22)	[20, 22, 23, 25, 27, 28, 32, 33, 37–39–41, 44, 46, 47, 49, 51, 53, 65, 67, 70]
Formal support - negative	31.9 (15)	[21, 22, 27, 28, 32, 37, 40, 42–44, 47, 52, 53, 65, 71]
Reluctance to engage in services	27.7 (13)	[22, 25, 28, 29, 32, 34, 37, 40–43, 46, 48]
**Practical changes**	**74.5 (35)**	[20, 22–26, 28, 29, 32, 33, 35–37, 39–49, 51, 54–57, 65–67, 70, 72, 73]
Living alone	31.9 (15)	[23, 25, 29, 32, 36, 37, 39, 41, 43–45, 47, 49, 65, 66]
Financial issues	25.5 (12)	[22, 28, 29, 32, 37, 39, 41, 44, 46, 47, 49, 70]
Caregiver health - negative	25.5 (12)	[20, 23, 26, 29, 32, 35, 36, 39, 41, 44, 45, 47, 49]
**Caregiver role**	**66.0 (31)**	[22–27, 31–37, 39–41, 43–49, 55–57, 65, 66, 70–72]
Constructing a new identity	44.7 (21)	[22–24, 26, 27, 31, 32, 34, 36, 37, 39–41, 44, 46–48, 55, 66, 70, 72]
Caregiver pride	19.1 (9)	[23, 35, 40, 41, 45, 48, 55, 56, 59]
Losing your identity	17.0 (8)	[26, 39, 41, 43–45, 47, 71]
Caregiver’s own mortality	17.0 (8)	[25, 33, 34, 41, 44, 46, 48, 66]
**Pre-loss help**	**53.2 (25)**	[22–24, 27, 29, 32, 33, 35, 40, 41, 43, 45–50, 53–57, 66, 67, 70]
doing their best	23.4 (11)	[22, 24, 32, 33, 41, 45, 46, 55–57, 67]
Caregiving general - positive	12.8 (6)	[27, 32, 33, 46, 53, 56]
Communication and patient attitude	12.8 (6)	[24, 35, 41, 45, 46, 48]
Patient-caregiver relationship	12.8 (6)	[24, 41, 43, 45, 47, 49]
Saying goodbye - positive	12.8 (6)	[23, 24, 27, 46, 50, 67]
**Pre-loss hinder**	**53.2 (25)**	[23, 24, 26, 27, 29, 32, 36, 39–41, 43–48, 50–53, 55, 56, 65–67, 70]
Caregiving general - negative	17.0 (8)	[24, 27, 41, 46, 51, 53, 56, 70]
Healthcare services - negative	14.9(7)	[32, 40, 41, 50, 52, 53, 70]
Saying goodbye - negative	12.8 (6)	[34, 38, 48, 49, 55, 70]
**Caregiver in context**	**34.0 (16)**	[23, 25, 30, 32, 33, 36, 38, 43, 45, 48–50, 54–57, 73]
Homophobia	10.6 (5)	[33, 38, 43, 54, 55]
Gender differences showing grief	8.5 (4)	[32, 48, 49, 56]
Stigma (HIV/AIDS)	8.5 (4)	[25, 33, 43, 55]
Caregiver language choices	8.5 (4)	[36, 43, 45, 57]
**Need for different supports**	**27.7 (13)**	[22, 24, 27–29, 32, 37, 42, 47, 50, 69, 70, 72]
Consistency	8.5 (4)	[27, 37, 42, 70]
Similar others	8.5 (4)	[28, 29, 32, 47]
Talking about loss	8.5 (4)	[22, 32, 70, 72]

Note: Bolded text indicates a theme, non-bolded a sub-theme

### Emotional journeys

This grouping of themes includes all mentions of the wide diversity of emotions and cognitive states that bereaved caregivers reported. Emotions were discussed in 42 of 47 studies, indicating that caregiver’s feelings were central to most investigations of their bereavement. Seven themes - broad domains of emotions and mental states - emerged from the literature. The theme most frequently discussed in the literature (35 studies) is here referred to as “serenity” and is characterized by uplifting feelings such as acceptance and relief. Caregivers’ experiences of serenity were sometimes momentary and sometimes long-lasting, and could correspond to small or large shifts in the caregiver’s sense of wellbeing. The other six themes drawn from the literature related to challenging, difficult, or painful emotions: sadness was reported in 34 studies, guilt and regret in 25, uncertainty in 25, trauma in 19, escape in 17 and anger in 15.

Sadness included feelings of loss and heartache, expressed through various terms such as depression, emptiness, and yearning. Feelings of guilt and regret were often related to caregiver’s perceptions of how their actions or inactions during caregiving affected the patient’s well-being. The theme of uncertainty was characterized by states such as confusion and ambivalence that could manifest as behaviors that were disorganized, passive, or distracted; this theme reflected an overall sense of uncertainty in the wake of a massive life change. Trauma included feelings and thoughts indicative of intense emotional turmoil, such as shock, obsessive thoughts, and consideration of suicide. The theme of escape pertained to caregivers’ efforts to avoid painful feelings, as well as the experience of numbness. Finally, the theme of anger contained feelings of anger, unfairness, and resentment that were directed at many targets including caregivers themselves, care receivers, the illness, healthcare professionals, family, or their faith in God.

It is important to note that while many of these emotions and states were not typically framed as desirable per se, they were not always deemed insufferable either, and were sometimes regarded as expected or accepted (for example, sadness was deemed to be necessary by one caregiver [20]). Caregivers can experience a combination of uplifting and challenging emotions, such as acceptance and pain [21–23] and sometimes conflicting emotions were joined - for example, in the case of relief and subsequent guilt [24, 25]. Negotiating these frequent emotional ups and downs was a key process and caregivers reported how spirituality and finding comfort in their faith was often helpful to finding emotional balance [26].

### Connecting with life again: Experiences of healing post-loss

This theme captures post-loss experiences reported to positively influence caregivers’ bereavement. Forty-one of 47 studies comprised this theme, indicating that nearly all of the surveyed literature addressed helpful post-loss events to some extent. The factors most frequently cited as helping caregivers cope with loss were positive experiences from receiving informal support from family and friends and formal support from professionals like social workers, support groups, and doctors. Different types of support (e.g. practical, emotional) were deemed important [27, 28], and even support from more peripheral contacts like neighbors [29] [30], and co-workers [31] was perceived as valuable. Support affirmed the importance of the caregivers’ relationship with the deceased [23], lessened feelings of overwhelm and defeat [32], and fostered caregivers’ motivation to continue living [33].

Individual coping strategies were the next most cited sources of comfort (reported in 18 studies), and these included behaviors like crying [31], using humor [34], taking 1 day at a time [35], and engaging in daily rituals like walks [36]. This theme also included caregivers’ efforts to honor and keep bonds with the deceased, and coping strategies that involved others (e.g. caregivers providing support to others) [29].

### Stumbling blocks: Post-loss experiences that disrupt the healing process

This theme is comprised of post-loss experiences that negatively influenced caregivers in their bereavement. In parallel with the finding that positive support from others was the most helpful post-loss experience, lacking or unhelpful support from family, friends, and formal sources such as therapists, was the post-loss experience most frequently associated with poor bereavement outcomes (family and friends cited in 22 studies and formal sources cited in 15 studies). Characteristics that made individuals less helpful sources of support included their lack of personal experience with death [37], their underestimation of the significance of the loss [25, 38], their pressure on the caregiver to “move on” [22, 27, 39], or conversely their pressure on the caregiver to talk about the loss when it was not desired [32].

In terms of formal supports, a general lack of follow-up services and information was reported across many studies; as one caregiver expressed “You go from having a whole army of people [while the patient is alive]; then it is just you” [21]. Unhelpful or lacking support led caregivers to feel helpless [33], invisible [40], or alienated from people in their lives [41].

Another factor found to negatively impact caregivers’ grieving process was intrinsic, rather than extrinsic: caregivers’ own reluctance to access bereavement support, mentioned in 13 studies. For some, this reluctance was present even before accessing services and was founded on skepticism regarding the usefulness of services [25, 29] perceived stigma surrounding mental health support [32, 37], or the belief that healing was a task one had to do alone [29, 42]. For others, this reluctance emerged after unhelpful experiences with professional support [22, 34, 37, 43]).

### The “work” after death: Practical tasks and lifestyle adjustments

This theme includes bereaved caregivers’ experiences of facing concrete tasks and lifestyle transitions after the loss of their care recipient that emerged in 35 studies. Living alone and completing household tasks alone (e.g. cooking for one, maintaining the garden) was the most commonly discussed adjustment for bereaved family caregivers. Caregivers reported losing not only companionship, but practical assistance their partners had provided, such as driving them to doctors’ appointments [39]. In one study, a caregiver expressed feeling as though they now have “to do it all” [44]. For some, this work was perceived to be a burden [28, 36, 44] that delayed or interrupted the grief process [41]. However, some individuals felt capable of making the adjustments that living alone demanded [45] and were proud of facing their new life “head-on” [40].

The loss of a partner was often associated with reduced income and financial stress (referenced in 12 studies), sometimes pressuring caregivers to return to work [37, 39]. Caregivers were also frequently thrust into financial tasks they had not previously been responsible for, like paying bills and filing taxes [22, 32, 46, 47].

Another detrimental impact that bereavement had on daily living was on caregivers’ physical health, as discussed in 12 studies. Although the physical work load carried during active caregiving was, to some extent, relieved after the loss, grief was often accompanied by exhaustion, disruptions to eating and sleeping habits, and the emergence or exacerbation of specific ailments like asthma [41].

### Performance of a lifetime: Caregivers’ appraisals of role and meaning

When caregivers lose their care receiver, their sense of their role and purpose in life may be impacted. In addition, caregivers’ sense of what their experiences meant to them may change. This theme pertains to caregivers’ evolving understandings of their role and the perceived meaning of their experiences. The sub-theme of constructing a new identity was most frequently discussed in the literature (in 21 studies) while the identity loss that preceded reconstruction was noted in 8 studies. Caregivers often described the loss of the identity they held while he or she was living, for example as a “wife,” “partner,” or “caregiver.” Individuals sometimes expressed feeling incomplete [41] or feeling as though they will never be their former self again [32]. The reconstructive acts that caregivers engaged in were discussed in various terms: transformation [22], reorganization [48], reinvention [37], and reinvestment in life [46]. Reconstruction involved making decisions about the kind of person the caregiver wanted to be, and the kind of life they wanted to begin leading after their loss.

Caregivers’ feelings of pride for their caring work was mentioned in 9 studies, and was framed as enhancing their positive regard for the whole experience as well as comforting them through their grief. Another prominent subtheme, found in 8 studies, was that of caregivers gaining a different sense of their own mortality following the loss. For some, this manifested as a greater awareness of their finitude, which sometimes encouraged positive changes in outlook or lifestyle [33, 41, 46]. For example, one caregiver expressed adopting a “do it now attitude” [41]. This awareness also prompted some caregivers to consider their end-of-life preferences [48]. Overall, confronting one’s own mortality was more often discussed in terms of positive impact rather than in terms of anxiety or crisis.

### A cloak of comfort: Resiliency-building before the loss

This theme emerged in 25 studies and includes caregiver characteristics, attitudes, and experiences of the pre-loss period reported to positively impact bereavement. The most common subtheme was that of having done one’s best (noted in 11 studies). Caregivers’ perception of having done their best for the patient was consistently described as lessening regret and relieving pain following the loss. Caregiving in general was also suggested to impact bereavement in 6 studies, and assisted one’s adjustment to loss if it had been perceived as a positive experience overall.

Communication between caregiver and care-receiver, along with the care-receiver’s attitude, comprised another common subtheme (found in 6 studies). Satisfactory communication meant “to enjoy the other’s presence despite the disease” [46] and to create positive memories that eased pain during the bereavement period, while open communication about the patient and caregivers’ sadness fostered a shared experience of grief leading up to the death [48]. The patient’s attitude could positively impact bereavement as well; for example, an attitude of gratefulness could instill the caregiver with an enduring sense of value [35], while an attitude of acceptance regarding death was encouraging and comforting even after the loss [46].

A subtheme closely tied to communication and patient attitude was that of caregiver-patient relationship, found in 6 studies. Many findings in this subtheme related to individuals who lost family members they were not very close to or felt enmity towards, for whom less closeness was thought to make the loss more bearable [41, 47, 49].

The nature of the caregivers’ good-bye to the patient was also found to impact post-loss experiences in 6 studies, with the general finding being that a satisfactory goodbye fostered positive grief outcomes and enhanced one’s capacity to carry on with life [23, 27, 46, 50]. Other subthemes of helpful pre-loss experiences included satisfactory relationships with health care staff, pre-planning for the loss, and the nature of the disease.

### The burden of before: Pre-loss experiences that negatively impact grief

This theme includes caregiver characteristics and experiences in the pre-loss period that were expressed as hindering or worsening coping abilities in bereavement. The work of caregiving in general was cited as detrimentally impacting bereavement in 8 studies. Participants and researchers did not suggest that caregiving in itself always leads to complications in grief, but instead that caregiving experiences perceived as disruptive and burdensome often contribute to difficulties post-loss. For example, when caregiving consumed individuals’ lives and left little time for them to connect with their social networks, they found themselves isolated from support in bereavement [51].

Another pre-loss experience found to make bereavement more difficult in 7 studies was negative interactions with healthcare services and providers. Perceived medical negligence [50], a lack of communication from health staff [52], and a lack of acknowledgement for the caregivers’ feelings and preferences [53], were some situations that contributed to caregivers’ feelings of anger and unrest towards health providers. These unresolved feelings sometimes preceded prolonged, traumatic grief [53]. Having an unsatisfactory goodbye with the care receiver was another pre-loss experience identified as damaging in 6 studies. Caregivers who missed their opportunity to witness final moments of their care recipient’s life expressed regret [50] and guilt [23]. Goodbyes which were extended over a long period of time (e.g. in the case of dementia) were identified as worsening caregivers’ grief as well [24].

### Caregiver in context: Social identities and sanctions

This theme explores the intersections between aspects of caregivers’ identities, their caregiving and bereavement experiences, and socio-cultural context. In 5 studies, sexual orientation was reported as an aspect of caregiver identity that impacted the grief experience. Homosexual individuals who cared for their partners described the homophobia that could impact support they received both prior to the patient’s death and after. For example, gay partners were not always granted the same hospital visitation privileges that family members were [33], and family members often assumed control of decisions about the fate of the patient’s body [38]and material possessions [43] after death. However, instances of the caregivers’ social network overcoming their homophobic feelings and offering support were also noted in the literature [54].

The next most prominent subtheme was stigma towards HIV and AIDS, with representation in 4 studies (3 in common with the first subtheme). Caregivers discussed assumptions that others made about patients’ diseases and their own health status, for example that the caregiver had contracted AIDS [43]. Expected or real reductions in support after disclosure of the patient’s infection were also noted [25, 55].

Another aspect of caregiver identity often noted as impacting the caregiving and bereavement experience was gender (emerging in 4 studies). Gender was found to impact both one’s internal experience of grief [56], one’s expression of grief [32, 48, 49], and the types of social support offered to the caregiver [30, 32]. A common finding was that cultural norms encouraged women to outwardly grieve, while discouraging men from the same.

The subtheme of caregiver language choices was found in 4 studies, and was comprised by instances of researchers noting patterns in caregivers’ verbal expressions. Although researchers did not always relate language choices to socio-cultural norms, this subtheme was grouped within this theme because discourse is culturally-bound. As examples, it was noted that caregivers often used depersonalizing language when referring to disease [45], and phrases that signal physical force when referring to the impact of their care recipient’s death [57].

### A need for different kinds of support

This theme pulls together the different kinds of support caregivers desired or appreciated in their time of bereavement, and emphasizes the unique needs and preferences of those facing loss. The types of support most frequently identified in the literature as helpful were consistent support, support from similar others, and opportunities for caregivers to talk about their loss. Consistent support was noted in 4 studies and meant support-provider continuity from pre- to post-bereavement. Often this continuity was desired or came from health care staff who had cared for the patient and established a relationship with the caregiver during the illness phase.

The subtheme of similar others was found in 4 studies and refers to individuals in the caregiver’s life who had experienced their own bereavement and could provide empathetic understanding. Similar others made caregivers feel as though there was not a time limit on their grief [32]. Some literature noted that caregivers valued support from those who had experienced the same kind of loss they had, e.g. the death of a partner [47].

The subtheme of talking about the loss was found in 4 studies and cited as a form of support that caregivers needed. The act of talking with another was construed as a way to release caregivers’ pain [39], and was reported to reduce caregiver anxiety in bereavement [32].

## Discussion

### Overarching findings

When all of the data from the whole sample was considered together, three overarching meta-themes were emerged. The three meta-themes we identified were: 1) a caregiver’s experiences during active caregiving can and does affect them into bereavement, 2) each caregiver’s experience of grief and loss is unique, and 3) there is a need for different kinds of supports. The meta-themes paint a broad picture of what caregivers experience and factors that impact them during their bereavement.

The themes reported the most often were “emotional journeys”, “connecting with life again”, “stumbling blocks”, and “the work after death”, indicating that these themes were most often mentioned as forming the experience of bereavement for family caregivers. The most prevalent theme focused on the range of emotions described by caregivers in bereavement, from serenity, acceptance and relief, to the very challenging, difficult, and painful emotions of sadness, guilt and regret, uncertainty, trauma, escape, and anger. This combination of emotions was expected and accepted and is reflected in the oscillation movement seen in Stroebe and Schut’s Dual-Process Model [58], where grief is often constantly fluctuating. Finding a balance between restoration-oriented and loss-oriented coping was associated with more positive bereavement outcomes [59]. The findings from this metasummary add to our understanding of the range of emotions and the normal responses that were deemed necessary to emerge from a very challenging life event such as the loss of most often, a life partner, as well as the caregiving role.

The second most common theme focused on the ways bereaved caregivers were connecting with life again, and what was helpful to them, mainly having support to lessen their negative feelings and motivate them to continue. The need for different kinds of support was described as bereaved caregivers often expressed how helpful a variety of supports (e.g. family, friend, and professional support) was to dealing with their grief. The participants did not always articulate the specific aspects of support that were appreciated (e.g. the empathy that came from others who had experienced similar bereavements), but having support was consistently emphasized. However, as every caring journey is unique – from the caregiver-patient relationship to the health care experiences to the nature and duration of the disease – it is essential that diverse supports be extended to individuals facing loss. This focus on the necessity of support is often missing from most theories of grief, where the individual tasks [60] become the main focus. More research is needed to identify tools and interventions to assess support and the most appropriate interventions [61] to offer. The third most common theme of “stumbling blocks” also related to support that negatively influenced caregivers in bereavement. Bereaved caregivers felt the significance of their losses were not recognized and they often described pressure to move on while being left without appropriate support.

Making practical and lifestyle adjustments constituted “the work after death” and the physical adjustments including exhaustion and disruption of sleep. Practitioners need to be aware that bereaved caregivers may present themselves in health care settings such as emergency rooms with physical needs that may arise from caregiving and grief. A careful assessment and identifying the underlying family situation will direct the most appropriate response that recognizes the bereaved caregiver’s unique needs. This relates to the individualized constructivist approach to finding meaning in bereavement, as described by the work of Neimeyer [62]; an approach that supports each caregiver’s unique narrative in personal, practical, or spiritual terms.

The “performance of a lifetime” described the bereaved caregivers’ need to make sense of their experiences and find meaning and for some, a new identity. The bereaved caregiver’s unique need for recognition of their experiences while in the caregiving role may be understood from the perspective of attachment to the deceased and the inner-focused, adaptive process of continuing bonds [63]. An interesting pattern emerging from the data is that pre-loss (active caregiving) experiences are discussed less frequently than post-loss experiences. Although it is understandable that bereavement researchers would attend mainly to the bereavement stage rather than the caregiving stage, a significant number of studies did affirm the importance of the pre-loss period in impacting a caregiver’s experience of grief. In a study involving both active and bereaved caregivers of persons with Motor Neuron Disease, experiences with palliative care services affected their ability cope with caregiving and not become overwhelmed [64]. As such, one of the meta-themes identified in the literature is the need to acknowledge and honor how the experiences of caregiving will affect the family caregiver in bereavement. This metatheme carries important implications for helping professionals across the spectrum of palliative care services, emphasizing the need to recognize and support the role of the family caregiver as well as invite bereaved caregivers to share not only their grief experience but their caregiving experiences as well.

### Limitations

The limitations of this research include the sample and the methods applied to analyse the studies included in this metasummary. The team was not able to locate qualitative research from developing or under-resourced countries and thus the findings are from those countries that do have established palliative care programs and supports in place. The methodology involves the frequency with which a particular topic is discussed and does not necessarily correspond to its level of importance in the caregivers’ life. The experiences and emotions that caregivers choose to express are, to some extent, responsive to the particular research questions or wonders that are posed in the researcher-participant encounter. While it is informative to aggregate and find similarities across findings reported by different inquiries, all themes emerging from analysis are valuable and capture different elements of caregivers’ lived experiences.

## Conclusions

For health care professionals such as nurses, physicians and social workers, the overarching findings provide a framework to inform practice and policy. Acknowledging the unique situation of the caregiver cautions against the danger of generalizing the bereaved caregiver experience. For example, a caregiver may be left emotionally and physically drained after caregiving and may be at elevated risk for complicated grief because of unresolved trauma they experienced. However, on the other hand a different caregiver may have found the caring experience to be a gift that bestowed him or her with a closer connection to the patient and a heightened appreciation for life. As explained in the first meta-theme, the experiences of caregiving, grieving the loss, and facing life without that person are connected chapters in individuals’ lives, and the challenges and joys of caregiving can and do create challenges and joys in bereavement. For example, knowing that a caregivers’ preferences were not honored by health care staff (while the patient was alive) could direct support professionals to query emotions like regret.

Recognizing that each caregiver’s experience is unique both before and after bereavement, emerged in all themes, as some caregivers experienced health improvement after the loss and others experienced physical deterioration. Another example of this uniqueness was that some caregivers felt a close relationship with the patient fostered positive bereavement outcomes and others felt a distanced relationship eased their grief. Moreover, caregivers described vastly different social support experiences after their loss. Caregivers were pained by different hurts and found resiliency through different sources.

The need for services and supports addressed specifically for family caregivers in their time of bereavement cannot be underestimated. Bereaved caregivers need a variety of supports that are helpful to them. Since each caregiver’s experience with caregiving and bereavement is unique, it follows that the supports that one caregiver finds helpful are different from the supports that another caregiver might find helpful. For example, some caregivers discussed finding comfort in their faith, however not all caregivers are spiritual and certainly not all caregivers practice their spirituality in the same way. Likewise, some caregivers found great support in formal bereavement services but other caregivers were reluctant to engage in such services. A careful assessment of family caregivers that begins during caregiving and extends in bereavement will identify those at most risk and interventions can be provided to prevent negative consequences such as complicated grief.

## Appendix 1

**Table 3:** Medline search strategy

1. adult children/ or caregivers/ or friends/ or exp. parents/ or siblings/ or spouses/
2. nuclear family/ or only child/ or siblings/ or spouses/
3. (carer or “care partner*” or partner or daughter or son or aunt or uncle or cousin or niece or nephew or grandchildren or granddaughter or grandson or neighbor* or neighbour*).mp. [mp = title, abstract, original title, name of substance word, subject heading word, keyword heading word, protocol supplementary concept word, rare disease supplementary concept word, unique identifier]
4. 1 or 2 or 3
5. exp. bereavement/
6. (grief or griev* or bereave* or sorrow* or misery* or mourn* or teariness or condole* or trauer).mp. [mp = title, abstract, original title, name of substance word, subject heading word, keyword heading word, protocol supplementary concept word, rare disease supplementary concept word, unique identifier]
7. (loss adj5 (grief or griev* or bereave* or sorrow* or misery* or mourn* or teariness or condole* or trauer)).mp. [mp = title, abstract, original title, name of substance word, subject heading word, keyword heading word, protocol supplementary concept word, rare disease supplementary concept word, unique identifier]
8. 5 or 6 or 7
9. (weight adj3 loss).mp. [mp = title, abstract, original title, name of substance word, subject heading word, keyword heading word, protocol supplementary concept word, rare disease supplementary concept word, unique identifier]
10. 4 and 8
11. 10 not 9
12. Palliative Care/
13. Hospice Care/
14. (“palliative care” or “palliative service*” or “palliative program*”).mp. [mp = title, abstract, original title, name of substance word, subject heading word, keyword heading word, protocol supplementary concept word, rare disease supplementary concept word, unique identifier]
15. (“hospice care” or “hospice service*” or “hospice program*”).mp. [mp = title, abstract, original title, name of substance word, subject heading word, keyword heading word, protocol supplementary concept word, rare disease supplementary concept word, unique identifier]
16. 12 or 13 or 14 or 15
17. 11 and 16
18. (((“semi-structured” or semistructured or unstructured or informal or “in-depth” or indepth or “face-to-face” or structured or guide) adj3 (interview* or discussion* or questionnaire*)) or (focus group* or qualitative or ethnograph* or fieldwork or “field work” or “key informant” or themes)).ti,ab. or interviews as topic/ or focus groups/ or narration/ or qualitative research/
19. 17 and 18
20. meta-analysis/
21. metasynthesis.mp.
22. meta-synthesis.mp.
23. meta synthesis.mp.
24. “review”/
25. 20 or 21 or 22 or 23 or 24
26. 19 not 25
27. limit 26 to (english language and humans and yr. = “1990 -Current”)

## Abbreviations

CASP: Critical Appraisal Skills Programme
PRISMA: Preferred Reporting Items for Systematic Reviews and Meta-Analyses
